# Compilation of a panel of informative single nucleotide polymorphisms for bovine identification in the Northern Irish cattle population

**DOI:** 10.1186/1471-2156-11-5

**Published:** 2010-01-25

**Authors:** Adrian R Allen, Malcolm Taylor, Brian McKeown, April I Curry, John F Lavery, Andy Mitchell, David Hartshorne, Rüdi Fries, Robin A Skuce

**Affiliations:** 1 Agri-Food and Biosciences Institute, Stoney Road, Belfast, Northern Ireland, BT4 3SD, UK; 2 Orchid Cellmark, 16 Blacklands Way, Abingdon Business Park, Abingdon, Oxfordshire, OX14 1DY, UK; 3 Lehrstuhl fuer Tierzucht, der Technischen Universitaet Muenchen, Alte Akademie 12, D-85354 Freising-Weihenstephan, Deutschland/Germany

## Abstract

**Background:**

Animal identification is pivotal in governmental agricultural policy, enabling the management of subsidy payments, movement of livestock, test scheduling and control of disease. Advances in bovine genomics have made it possible to utilise inherent genetic variability to uniquely identify individual animals by DNA profiling, much as has been achieved with humans over the past 20 years. A DNA profiling test based on bi-allelic single nucleotide polymorphism (SNP) markers would offer considerable advantages over current short tandem repeat (STR) based industry standard tests, in that it would be easier to analyse and interpret. In this study, a panel of 51 genome-wide SNPs were genotyped across panels of semen DNA from 6 common breeds for the purposes of ascertaining allelic frequency. For SNPs on the same chromosome, the extent of linkage disequilbrium was determined from genotype data by Expectation Maximization (EM) algorithm. Minimum probabilities of unique identification were determined for each breed panel. The usefulness of this SNP panel was ascertained by comparison to the current bovine STR Stockmarks II assay. A statistically representative random sampling of bovine animals from across Northern Ireland was assembled for the purposes of determining the population allele frequency for these STR loci and subsequently, the minimal probability of unique identification they conferred in sampled bovine animals from Northern Ireland.

**Results:**

6 SNPs exhibiting a minor allele frequency of less than 0.2 in more than 3 of the breed panels were excluded. 2 Further SNPs were found to reside in coding areas of the cattle genome and were excluded from the final panel. The remaining 43 SNPs exhibited genotype frequencies which were in Hardy Weinberg Equilibrium. SNPs on the same chromosome were observed to have no significant linkage disequilibrium/allelic association. Minimal probabilities of uniquely identifying individual animals from each of the breeds were obtained and were observed to be superior to those conferred by the industry standard STR assay.

**Conclusions:**

The 43 SNPs characterised herein may constitute a starting point for the development of a SNP based DNA identification test for European cattle.

## Background

### Animal & Food Traceability - Conventional Approaches and Problems

The identification and registration of livestock and monitoring of their movements is an essential part of agricultural policy for national governments. Such schemes underpin disease control, grant and subsidy management, food hygiene/safety assurance and facilitate product recalls if necessary.

As a result of these benefits, and also in response to major animal health problems such as BSE (Bovine Spongiform Encephalopathy) and Foot and Mouth Disease that impact livestock, producers and consumers, many countries in the developed world have adopted national databases, based on numbered ear tags, to record cattle identity and movements [1,2].

Trade globalisation has also strengthened the case for improved animal traceability. Modern consumers confronted with increased choice from multiple sources may also face elevated risks as a result of increased likelihood of chemical/pathogenic contamination in foodstuffs [3,4]. Such crises can compromise the economic well being of agri-food industries as well as affecting the health and confidence of the consumer. The BSE crisis of the 1990s aptly demonstrates both points and proves that the actions of one state can impact negatively on the public health of others [5].

In light of the potential dangers highlighted above, the European Union (EU) passed Council Directive 92/102/EEC stating that all bovine animals in Member States should be identified with an ear-tag bearing a unique identification code [6]. The more far reaching Council Regulations 1760/2000 and article 18 of regulation 178/2002 [7,8], established the need for Member States to control registration and tracking of bovine animals through a computerised database based on a two ear-tag system. These enhanced traceability regulations also extended to the products derived from the animal after slaughter, insisting that batches of meat must be able to be tracked back to animal's of origin by use of carcase, primal and pack labelling. These regulations were designed to ensure that full animal and meat traceability was "established at all stages of production, processing and distribution".

Future development of tag schemes may include the use of electronic identification devices such as radio frequency identification (RFID) ear-tags, ruminal boluses and injectable transponders which automate the reading of animal identity, thereby reducing transcription errors [9].

Whilst lauding the ability of such systems to improve farm management, a recent EU report on uses of electronic identification in the agri-food industries (the IDEA project - IDentification Electronique des Animaux - http://idea.jrc.it/) confirmed that the preferred option, RFID ear-tags, still suffered from the same accidental tag loss and fraudulent switching problems which afflict conventional schemes [10].

Animal mis-identification resulting from tag loss has profound epidemiological and traceability implications which can result in costly consequences for herd keepers. The extent of tag loss and its negative impact on the ability to re-assign correct ID has been highlighted in a recent study in intensively farmed buffalo, which revealed mean conventional ear-tag retention time to be 272 days [11]. Similarly, deficiencies in meat labelling at abattoirs and at retail outlets have resulted in loss of correct association between batch numbers and samples. A recent study used DNA traceability to indicate that 2% of randomly selected samples from labelled carcases at the abattoir did not match the profiles of the animals they were purported to come from. This increased to 3% when sampling was conducted at the point of sale [12].

The root of these problems in conventional and electronic tagging/labelling systems arises from their reliance on methods which track devices attached to animals and their products, but not the animals or products themselves. However, DNA profiling, which utilises unalterable biological properties of individual animals to produce a unique identifier, offers a potential solution for scientifically verifying animal identity [13]. At present the technology does not exist for DNA profiles to be read in 'real time', unlike tags and labels. This limits DNA's use as a primary identifier of animals and derived food products. However, despite these limitations it can be used effectively in retrospective audits to verify tag identity and quality assure existing meat tracing technologies [1,3,14,15]. The added advantage of DNA based parentage verification makes it a powerful method for verifying national herd database information.

### DNA markers and DNA Profiling

The level of polymorphic variation observed in bovine short tandem repeats (STRs) permits the use of relatively few loci to identify cattle and to determine their parentage [16,17]. Several well defined cattle STR panels are commercially available. One such 11 marker panel has been approved by the International Society of Animal Genetics (ISAG) for use by breed societies to register pedigree sires and dams and their progeny.

Single Nucleotide polymorphisms (SNPs) are a much simpler form of DNA variation than microsatellites, involving a single nucleotide change at one position of the genetic code. This lack of diversity makes SNP markers less informative than microsatellites. As a result, single SNPs don't discriminate between individuals very well. However, genotyping of multiple SNPs can overcome this problem [18].

SNP genotyping also has distinct advantages over STR genotyping. Owing to their smaller size, they are less prone to gametic mutation and therefore do not suffer from the same non-identical by descent problems that can affect STRs as they mutate between generations [18]. SNPs are also more robust in their interpretation/analysis than STRs [19] and can be genotyped by a variety of high throughput methods on various platforms, many of which can be automated with economy of scale savings [20]. With the advent of ever-improving genotyping technologies [21], it has recently become attractive to develop a SNP-based DNA profiling system for the identification of cattle. Two SNP panels have recently been assembled for this purpose [18,22].

### Cattle Farming in Northern Ireland

There are approximately 1.7 million cattle in Northern Ireland spread over 25000 herds with an average herd size of seventy animals. The Department of Agriculture and Rural Development (DARD) runs a computer database called the Animal and Public Health Information System (APHIS) based on the two ear tag system required by the European Commission [7,8]. This system manages identification, registration, movement, testing, subsidy payment and general traceability of animals. To date, APHIS facilitates the registration of all animal births, movements, test histories and slaughter [1].

### Study Aims

We attempted to assemble a panel of SNPs which would identify animals from the Northern Irish population. SNP panels previously identified by Heaton et al., 2002 and Werner *et al*., 2004 [18,22] were characterised in our cattle population to determine which would uniquely identify bovine animals. An allele frequency study was carried out in representative breed panels of the most common breeds used in Northern Ireland. This work was undertaken using a semen archive of sires from each of the six predominant cattle breeds, unrelated at the parent and grandparent level.

For validation purposes, it was desirable to compare the capability of any new SNP panel against the existing industry standard test. We STR genotyped a representative subset of the Northern Irish Cattle population using the ISAG approved Stockmarks II system. A general random sampling across the whole province that included many breeds was considered sufficient to determine allele frequencies for each locus and subsequently to help determine the minimum probability of finding two animals that possessed the same STR profile. Ultimately this would enable us to ascertain the uniqueness of STR profiles in our population, vital information for deployment of this technology as a counter fraud and traceability tool.

## Results

Allele frequencies of the 51 SNPs genotyped across the semen DNA panels from six breeds are listed in the Additional file 1 Table S1). Six SNPs which exhibited minor allele frequency less than 20% in more than three of the breed panels were excluded (16_2, 448_67, 487_67, Bulge101, MBS047-1 and 454_G11).

Allele frequencies of the remaining 45 SNPs were used to calculate expected genotype frequencies in each of the breed panels. Chi squared analysis of expected and observed genotype frequencies revealed no significant difference (p > 0.05) for any of the SNPs. This indicates that in each of the breed panels, all SNPs are in Hardy Weinberg Equilibrium (HWE) (Additional file 1 Table S1).

Exact chromosomal locations including contig accession numbers for each of the 45 remaining SNPs on the current build of the bovine genome were determined by the NCBI's BLAST (Basic Local Alignment Search Tool) (Table 1). Two further SNPs were excluded because they encoded amino acids in proteins and were not deemed to be selectively neutral (MBS030-1 and MBS031-1) (Table 1).

**Table 1:** SNP id, chromosome and contig locations including Linkage Disequilibrium between SNP pairs on the same chromosome. D' was calculated by EM algorithm between pairs of SNPs in adjacent order

SNP Name	Chromosome	**Contig Accession no**.	Base position in Contig	Genbank SNP id	Is SNP coding or non coding?	Combined Breeds Linkage Disequilibrium (D')
027.sp6	1	[Genbank: NW_001493890]	3818758	ss_182258874	Non coding intronic	027.sp6 - 421_10 D' = 0.18
	
421_10	1	[Genbank: NW_001493804]	583808	ss_182258864	Non coding intronic	

MBS029-1	2	[Genbank: NW_001494698]	3047667	rs17871918	Non Coding	MBS029-1 - MBS042-1 D' = 0.02
	
MBS042-1	2	[Genbank: NW_001494591]	108095	rs41257512	Non coding intronic	

018.sp6	3	[Genbank: NW_001494745]	2606666	ss_182258872	Non coding intronic	018.SP6 - 486_67 D' = 0.41
	
486_67	3	[Genbank: NW_001494789]	1283668	ss_182258886	Non Coding	

417_16	4	[Genbank: NW_001494939]	1975758	ss_182258885	Non Coding	417_16 - MBS048-1 D' = 0.12
	
MBS048-1	4	[Genbank: NW_001494921]	1019822	rs17871345	Non coding intronic	

431_A2	5	[Genbank: NW_001495040]	1991472	ss_182258884	Non Coding	Exclude SNP MBS030-1 MBS007-1 - 431_A2 D' = 0.09
	
MBS007-1	5	[Genbank: NW_001495013]	223977	rs41256853	Non coding intronic	MBS007-1 - MBS043-1 D' = 0.06 431_A2 - MBS043-1 D' = 0.12
	
MBS030-1	5	[Genbank: NW_001494990]	1339300	rs17871971	Coding - first base in R codon	
	
MBS043-1	5	[Genbank: NW_001495111]	1628598	rs29003967	Non coding intronic	

013.sp6	6	[Genbank: NW_001495169]	651244	ss_182258871	Non coding intronic	013.sp6 - AH2-5 D' = 0.20
	
AH2-5	6	[Genbank: NW_001495205]	919203	rs41255759	Non coding intronic	

MBS044-1	7	[Genbank: NW_001495330]	1925089	rs17870555	Non coding intronic	N/A

128.sp6	8	[Genbank: NW_001495453]	6360496	ss_182258880	Non coding intronic	N/A

004.sp6	9	[Genbank: NW_001495578]	257732	ss_182258869	Non coding	423_24 - 425_2 D' = 0.48 423_24 - 004.sp6 D' = 0.10
	
423_24	9	[Genbank: NW_001495569]	793596	ss_182258865	Non Coding	425_2 - 004.sp6 D' = 0.04

425_2	9	[Genbank: NW_001495569]	839251	ss_182258866	Non coding intronic	

022.t7	10	[Genbank: NW_001492799]	128540	ss_182258873	Non coding intronic	Exclude SNP MBS031-1
	
MBS031-1	10	[Genbank: NW_001492799]	1124499	rs17871372	Coding - third base in C codon	

055.t7	11	[Genbank: NW_001492961]	1632290	ss_182258877	Non coding	AH8-4 - 055.t7 D' = 0.02 AH8-4 - MBS015-1 D' = 0.21
	
AH8-4	11	[Genbank: NW_001492942]	738417	rs41255717	Non coding intronic	055.t7 - MBS015-1 D' = 0.09
	
MBS015-1	11	[Genbank: NW_001493001]	1324153	rs17871661	Non coding intronic	

058.sp6	12	[Genbank: NW_001493063]	1152348	ss_182258883	Non coding	N/A

AH25-1	13	[Genbank: NW_001493148]	1355813	rs41257524	Non Coding	AH25-1 - MBS046-1 D' = 0.20
	
MBS046-1	13	[Genbank: NW_001493168]	1973152	rs41257490	Non coding intronic	

436_C10	14	[Genbank: NW_001493182]	723654	ss_182258882	Non Coding	N/A

Bulge105	16	[Genbank: NW_001493378]	486637	ss_182258868	Non coding intronic	N/A

MBS018-1	17	[Genbank: NW_001493523]	2800849	rs41255724	Non coding intronic	N/A

105.sp6	18	[Genbank: NW_001493559]	10050072	ss_182258881	Non coding	105.sp6 - MBS033-1 D' = 0.07 105.sp6 - MBS021-1 D' = 0.41
	
MBS021-1	18	[Genbank: NW_001493612]	23880	rs17871403	Non Coding	MBS033-1 - MBS021-1 D' = 0.07
	
MBS033-1	18	[Genbank: NW_001493607]	941203	rs17871744	Non coding intronic	

039.t7	19	[Genbank: NW_001493666]	1151813	ss_182258875	Non coding	039.t7 - MBS054-1 D' = 0.11
	
MBS054-1	19	[Genbank: NW_001493688]	831437	rs41257458	Non coding intronic	

007.sp6	20	[Genbank: NW_001493983]	863397	ss_182258870	Non coding intronic	Bulge113 - 007.sp6 D' = 0.18
	
Bulge 113	20	[Genbank: NW_001493954]	1202924	ss_182258867	Non Coding	

048.sp6	21	[Genbank: NW_001494027]	363121	ss_182258876	Non coding intronic	N/A

MBS025-1	23	[Genbank: NW_001494145]	446755	rs41255852	Non coding intronic	MBS025-1 - MBS035-1 D' = 0.05
	
MBS035-1	23	[Genbank: NW_001494172]	276736	rs17872223	Non coding intronic	

MBS028-1	24	[Genbank: NW_001494262]	534627	rs17870274	Non coding intronic	N/A

070.t7	25	[Genbank: NW_001494320]	325234	ss_182258878	Non coding	MBS040-1 - 070.t7 D' = 0.05

MBS040-1	25	[Genbank: NW_001494295]	390532	rs17872131	Non coding intronic	

090.t7	28	[Genbank: NW_001501727]	3153055	ss_182258879	Non coding	N/A

MBS041-1	29	[Genbank: NW_001494538]	322380	rs17870855	Non coding intronic	N/A

Several of the remaining 43 SNPs were observed to exist on the same chromosome. Genotype data for SNPs located on the same chromosomes were collated from each of the six breeds studied, and subjected to EM algorithm analysis to determine the degree of allelic association/linkage disequilbirum (LD) between polymorphisms (Table 1).

The frequencies of the most common 43 SNP genotypes from each of the six breeds were then collated to determine the minimal probability of uniquely identifying unrelated animals from each breed. These probabilities ranged over three orders of magnitude from 2.45 × 10^-11 ^to 4.65 × 10^-13 ^(Table 2).

**Table 2:** Minimal probabilities of unique identity conferred by the 43 SNP panel and 11 STR assay.

Marker Type	Study Population	Probability of Identity
11 STR assay	NI representative sampling	1.89 × 10^-9^

43 SNP assay	A. Angus semen archive	2.45 × 10^-11^

43 SNP assay	Belgian Blue semen archive	3.31 × 10^-12^

43 SNP assay	Charolais semen archive	6.17 × 10^-12^

43 SNP assay	Holstein semen archive	4.65 × 10^-13^

43 SNP assay	Limousin semen archive	1.96 × 10^-12^

43 SNP assay	Simmental semen archive	2.36 × 10^-12^

Allele frequencies of the eleven STRs tested in the representative sampling of the Northern Irish cattle population are listed in Additional file 2 Table S2. Expected genotype frequencies were calculated from allele frequency data and compared by Chi squared analysis against observed genotype frequencies. This result revealed no significant difference (p > 0.05) between observed and expected genotypes (Additional file 2 Table S2) indicating that each locus is in HWE. The minimal probability of uniquely identifying an individual animal using the STR markers was determined to be 1.89 × 10^-9 ^(Table 2).

## Discussion

In selecting a panel of SNPs which would uniquely identify Northern Irish animals we were presented with several novel problems. Initially, which SNPs should be selected? Some of the best characterised SNPs at the inception of this work were those described by Werner *et al*. and Heaton *et al*., [18,22]. However unlike the Stockmarks STR based system, these SNPs were not well characterised across a variety of cattle breeds from different countries. The Northern Irish cattle population, whilst possessing many of the same breeds as those seen in Europe and the USA, may have been subject to region-specific population changes and inbreeding which could result in SNP allele frequencies slightly different than those observed in other countries. Therefore, it is plausible that a panel that is useful in one population may not necessarily be as useful in another [19]. As a result, it was essential to perform an allele frequency study for each SNP in our population to ensure that non-informative SNPs (minor allele frequency less than 20%) were not used in the final panel. Within populations of cattle there will also be differences in observed allele frequencies between breeds, just as is seen in sub-groups of different race/ethnicity in the human population. Indeed recent work by the Bovine HapMap consortium has illustrated the great diversity that exists between breeds and the conversely small diversity within breeds. This is indicative that the taurine ancestors of all modern *Bos taurus *breeds were very genetically diverse post-domestication, and that subsequent founder effects at time of breed formation and resulting small effective population size caused reduced diversity within breeds [23]. Therefore, we endeavoured to determine allele frequencies of the candidate SNPs in six of the most commonly seen breeds in Northern Ireland. Only 6 of the 51 SNPs studied were observed to exhibit minor allele frequencies of lower than 20% in three or more of the six breeds studied. This indicates that the vast majority of the panel described herein is suitable for use in identifying cattle of the major breeds found in Northern Ireland.

The fact that all SNPs were in HWE is indicative that random genetic drift and selection have not acted on these loci, or on loci in linkage disequilibrium with them and that they are subsequently stable markers, which can be used as identifiers.

SNPs used for identification purposes should ideally come from non-coding parts of the bovine genome since these regions are less likely to be influenced by natural or artificial selection, which could alter their allelic frequency over a period of time. SNPs affected by selection would not be sufficiently stable between generations to act as identifiers. MBS030-1 and MBS031-1 were excluded from the final SNP panel because they reside in coding regions of the bovine genome. Whilst HWE is observed for these SNPs in all tested breeds, indicating that selection is not playing a role, it was decided to exclude them since it was feasible that at some stage in the future a selective event could conceivably affect allele frequency.

Finally, to further maximise the discriminatory power of each individual SNP it was necessary to ensure that those occurring on the same linkage unit/chromosome, did not exhibit strong allelic association/linkage disequilibrium in this population. Allelic association between SNPs would render one of a pair completely uninformative as an identifier. Whilst Werner *et al *and Heaton *et al *had positioned their respective panels on chromosomes using fluorescence in situ hybridisation and linkage mapping respectively, only Heaton *et al *[22] had calculated the LD between SNPs on the same chromosomes. Quantification of LD between SNPs from both of these panels had not been undertaken. LD in the bovine genome has been observed to extend over large distances (1 Mb) with an average haplotype block size of between 26 and 113 kb [24]. This extent of LD over large distances exceeds that observed in humans and is caused by reduced allelic diversity resulting from small effective population sizes as a consequence of breed formation bottlenecks [23,24]. The latter also highlights the fact that spacing of potential identification markers very far apart is necessary to ensure maximum discriminatory power. In this study, we benefitted from the recent completion of the bovine genome and were able to exactly place the SNPs studied onto contigs of the sequenced bovine chromosomes by use of BLAST sequence alignment software on the NCBI website. LD calculation by EM algorithm indicated that for all SNPs sharing chromosomes, the values of D' were low. Subsequent Haploview analysis of these D' values and their confidence bounds revealed that they lie outside the default D' confidence interval (upper bound 0.98; lower bound 0.70) defined by Gabriel *et al*. as constituting a tightly bound block of high allelic association [25]. These data are indicative that the alleles of SNPs residing on the same chromosomes can segregate independently of one another, thereby retaining their informativity as unique identifiers.

The probabilities of the unique 43 SNP-based identity determined for each of the breeds in this study are comparable to those described by Werner *et al *and Heaton *et al *for a comparable number of SNPs. In reality, within pedigree and non pedigree herds, there is a greater reliance on breeding with the same sires and dams, and consequently a higher level of relatedness between animals. This reduces the allelic diversity greatly within such herds and subsequently increases the probability that two individual animals sired in different matings of the same sire/dam pair can possess the same SNP derived DNA profile. Consequently, it may require the genotyping of a few additional SNPs in such related animals to uniquely identify them. The bovine HapMap Consortium's data on a genome wide survey of SNP variation across several cattle breeds [23] takes account of the degree of relatedness between animals of the same breed, and indicates that approximately 50 SNPs should be used to determine the identity of animals and uniquely identify them with a match probability of 1.3 × 10^-15^.

In comparison to the commercially available ISAG approved set of STR markers, the potential discriminatory power of this SNP panel is significantly greater. Providing a suitable platform can be found to run the SNPs described in multiplex, the benefits of a next generation SNP-based DNA profiling system are substantial. Running costs could be reduced and analysis made simpler. Inter-laboratory comparisons and standardisation would be easier to achieve as a result of the bi-allelic simplicity of SNPs compared to more polymorphic STRs. Future test panels could also incorporate the growing number of SNP markers associated with desirable phenotypes/quantitative traits.

In Northern Ireland, cattle DNA profiling has been evaluated in several scenarios assure the existing tag based method of identification. These include the auditing of meat plants involved in processing over thirty month animals for TSE (Transmissible Spongiform Encephalopathy) surveillance, quality assurance of removal of tuberculosis and brucellosis infected animals from affected herds and investigations into alleged fraudulent identity swapping. The technique has also been deployed to aid farmers in re-establishing the identity of animals which have lost ear-tags and subsequently fall foul of animal identification requirements. In future, it is conceivable that DNA profiling could be used as a means of auditing the entire APHIS system in a way which is more powerful than any paper based audit. It is also foreseeable that such a traceability system could expand to become an effective means of tracking animals and their products from 'gate to plate'. These developments would greatly enhance the traceability capability of the existing system and lead to greater consumer and producer confidence.

## Conclusions

In recent times, international consortia have been moving towards configuration of a high throughput SNP based assay for cattle identification and parentage verification purposes. We believe that whilst more SNPs could be added to increase the discriminatory power between closely related individuals, the panel characterised here serves as a good starting point for many of the major European cattle breeds.

## Methods

### Sample sets

Semen samples were collected for the six major cattle breeds used in Northern Ireland - Limousin (*n *= 37), Belgian Blue (*n *= 35), Simmental (*n *= 32), Charolais (*n *= 34), Aberdeen Angus (*n *= 38) and Holstein (*n *= 29). Pedigree information was used to ensure that genotyped animals from each breed panel were unrelated at the parent and grandparent levels.

From statutory annual testing of all females and breeding bulls over twelve months of age in the national herd, a randomly selected sub-sample of clotted blood from 366 animals from across Northern Ireland's ten Divisional Veterinary Offices (DVO) were selected for inclusion in a population representative sample set collected over a 6 month period. This sample set provided a confidence level of 95.0% that allelic frequencies observed were representative of the greater national bovine population. Numbers of animals per breed are shown in Table 3.

**Table 3:** Breed breakdown of random Northern Ireland cattle population sampling.

Cattle Breed	**No**.
Aberdeen Angus*	34

Belgian Blue*	33

Blonde D'Acquitaine	16

Charolais*	73

Dexter	3

Hereford	11

Holstein*	28

Jersey	1

Limousin*	112

Parthenaise	1

Salers	4

Shorthorn	8

Simmental*	42

Total:	366

* Breeds used in SNP allele frequency study.

### DNA Extraction

DNA was extracted from both clotted blood and semen samples using Qiagen QIAamp DNA Mini kits (Cat no. 51306) following manufacturers instructions.

### PCR and analysis

#### SNP

DNA derived from semen (50 ng) was subjected to single-plex PCR for each of the SNPs genotyped. 20% of samples were genotyped a second time for quality control. PCR reactions were carried out in 25 μl total volume, 2 mM MgCl_2_, 0.2 μM of each primer, 2 mM of each dNTP, 10 mM Tris-HCl, 50 mM KCl, 0.5 U of Qiagen HotStar Taq polymerase. The cycling conditions used were an initial 96°C for 15 minutes to activate the hot start Taq, followed by 35 cycles of 30 s denaturation at 96°C, 1 min 30 s annealing at 53°C and 1 min 30 s extension at 72°C. Amplification products were SNP genotyped by pyrosequencing [26]. Genbank accession numbers of sequences containing all SNPs and location of SNPs are available [18,22].

#### STR

DNA derived from clotted blood (50 ng) was subjected to multiplex PCR at the eleven microsatellite markers in the Applied Biosystem's Bovine Stockmarks II assay (Cat no. 4307480) as suggested by the manufacturer. Amplified products were resolved by gel capillary electrophoresis on an Applied Biosystems ABI3100 Genetic Analyser. Analysis and allele calling was performed using Applied Biosystems Gene-Mapper software.

#### Statistics

The NCBI bovine genome BLAST tool was utilised to determine the exact physical location of each of the SNP markers in the recent draft of the bovine genome, Btau 4. Using Haploview [27], EM algorithm was then applied to SNP genotype data to determine the level of linkage disequilibrium (D') observed between markers on the same chromosome. Subsequent comparison of observed D' confidence bounds to a default D' confidence interval, defined as being indicative of significant allelic association by Gabriel *et al *[25], was undertaken using Haploview [27]. This permitted determination of whether pairs of SNP alleles analysed displayed significant levels of linkage disequilibrium/allelic association, or were able to segregate independently.

Allele frequency data was used to determine population genotype frequencies for both sets of markers. Chi squared analysis of expected and observed genotype frequencies was undertaken to ensure that alleles observed were in HWE. Most common genotype frequencies at each marker were then determined. These most common genotype frequencies were then multiplied together and used to determine the minimal probability of two randomly drawn animals from the same population possessing the same SNP or STR profile [14].

## Authors' contributions

AA designed and performed SNP genotyping assays, participated in the study design determined allele frequencies, performed statistical analysis and drafted the manuscript. MT participated in study design and genotyping of STR markers. BMcK participated in study design and contributed to SNP genotyping. AC designed and performed SNP genotyping assays, contributed to databasing of results and oversaw extraction of DNA from blood and semen samples. JL performed STR genotyping and databasing of results and oversaw extraction of DNA from blood and semen samples.

AM and DH contributed to study design. RF contributed to study design and provided SNP sequence details. RS conceived of the study and contributed to study design. All authors have read and approved the final manuscript.

## Supplementary Material

Additional file 1 **SNP allele frequency data**. All SNP allele frequency data for each of the six breeds studied are contained in this table along with Hardy Weinberg chi square p values, most common genotypes and most common genotype frequency. Click here for file

Additional file 2 **STR allele frequency data**. All STR allele frequency data for the NI cattle population representative random sampling are contained in this table along with Hardy Weinberg chi square p values, most common genotypes and most common genotype frequency. Click here for file
