# Supplementary material for: Compilation of a panel of informative single nucleotide polymorphisms for bovine identification in the Northern Irish cattle population
Source: BMC Genet. 2010 Jan 25;11:5. doi: 10.1186/1471-2156-11-5 (PMC2826282; doi:10.1186/1471-2156-11-5)
Supplement: Additional file 1 — SNP allele frequency data. All SNP allele frequency data for each of the six breeds studied are contained in this table along with Hardy Weinberg chi square p values, most common genotypes and most common genotype frequency. [file 1471-2156-11-5-S1.DOC]

| **SNP Name** | **SNP Reference** | **Breed** | **Minor Allele** | **Allele Frequency** | **Hardy Weinberg P value** | **Most Common Genotype** | **Genotype Frequency** |
| --- | --- | --- | --- | --- | --- | --- | --- |
| 027.sp6 | Werner *et al.,* (2004) | Aberdeen Angus | C | 0.26 | 0.999 | TT | 0.55 |
|  |  | Belgian Blue | C | 0.36 | 0.931 | CT | 0.46 |
|  |  | Charolais | T | 0.48 | 0.884 | CT | 0.50 |
|  |  | Holstein | C | 0.36 | 0.999 | CT | 0.46 |
|  |  | Limousin | T | 0.43 | 0.893 | CT | 0.49 |
|  |  | Simmental | T | 0.47 | 0.881 | CT | 0.50 |
| 421_10 | Werner *et al.,* (2004) | Aberdeen Angus | G | 0.47 | 0.784 | GC | 0.50 |
|  |  | Belgian Blue | G | 0.36 | 0.931 | GC | 0.46 |
|  |  | Charolais | G | 0.17 | 0.999 | CC | 0.69 |
|  |  | Holstein | C | 0.43 | 0.991 | GC | 0.49 |
|  |  | Limousin | G | 0.21 | 0.837 | CC | 0.63 |
|  |  | Simmental | G | 0.28 | 0.680 | CC | 0.52 |
| 018.sp6 | Werner *et al.,* (2004) | Aberdeen Angus | T | 0.28 | 0.856 | CC | 0.52 |
|  |  | Belgian Blue | C | 0.20 | 0.999 | TT | 0.65 |
|  |  | Charolais | T | 0.35 | 0.858 | TC | 0.46 |
|  |  | Holstein | C | 0.43 | 0.938 | TC | 0.49 |
|  |  | Limousin | C | 0.41 | 0.797 | TC | 0.48 |
|  |  | Simmental | T | 0.23 | 0.668 | CC | 0.60 |
| 486_67 | Werner *et al.,* (2004) | Aberdeen Angus | T | 0.36 | 0.880 | TC | 0.46 |
|  |  | Belgian Blue | T | 0.11 | 0.770 | CC | 0.79 |
|  |  | Charolais | T | 0.14 | 0.589 | CC | 0.74 |
|  |  | Holstein | T | 0.16 | 0.773 | CC | 0.71 |
|  |  | Limousin | T | 0.23 | 0.779 | CC | 0.59 |
|  |  | Simmental | T | 0.30 | 0.829 | CC | 0.49 |
| 417_16 | Werner *et al.,* (2004) | Aberdeen Angus | G | 0.08 | 0.579 | AA | 0.84 |
|  |  | Belgian Blue | G | 0.41 | 0.614 | AG | 0.48 |
|  |  | Charolais | G | 0.45 | 0.999 | AG | 0.50 |
|  |  | Holstein | G/A | 0.50 | 0.572 | AG | 0.50 |
|  |  | Limousin | G | 0.30 | 0.859 | AA | 0.49 |
|  |  | Simmental | A | 0.30 | 0.874 | GG | 0.49 |
| 431_A2 | Werner *et al.,* (2004) | Aberdeen Angus | G | 0.21 | 0.493 | AA | 0.63 |
|  |  | Belgian Blue | A | 0.40 | 0.942 | GA | 0.48 |
|  |  | Charolais | A | 0.33 | 0.967 | GG | 0.45 |
|  |  | Holstein | G | 0.41 | 0.864 | GA | 0.48 |
|  |  | Limousin | A | 0.30 | 0.568 | GG | 0.49 |
|  |  | Simmental | G | 0.48 | 0.746 | GA | 0.50 |
| 013.sp6 | Werner *et al.,* (2004) | Aberdeen Angus | C | 0.26 | 0.259 | TT | 0.55 |
|  |  | Belgian Blue | T | 0.47 | 0.598 | CT | 0.50 |
|  |  | Charolais | T | 0.45 | 0.883 | CT | 0.50 |
|  |  | Holstein | C | 0.37 | 0.912 | CT | 0.47 |
|  |  | Limousin | C/T | 0.50 | 0.999 | CT | 0.50 |
|  |  | Simmental | T | 0.37 | 0.999 | CT | 0.47 |
| 128.sp6 | Werner *et al.,* (2004) | Aberdeen Angus | A | 0.47 | 0.964 | GA | 0.50 |
|  |  | Belgian Blue | A | 0.16 | 0.999 | GG | 0.71 |
|  |  | Charolais | A | 0.33 | 0.915 | GG | 0.45 |
|  |  | Holstein | G | 0.34 | 0.828 | GA | 0.45 |
|  |  | Limousin | A | 0.30 | 0.999 | GG | 0.49 |
|  |  | Simmental | A | 0.39 | 0.999 | GA | 0.48 |
| 004.sp6 | Werner *et al.,* (2004) | Aberdeen Angus | G | 0.49 | 0.652 | GA | 0.50 |
|  |  | Belgian Blue | A | 0.33 | 0.999 | GG | 0.45 |
|  |  | Charolais | A | 0.32 | 0.835 | GG | 0.46 |
|  |  | Holstein | G | 0.33 | 0.840 | AA | 0.45 |
|  |  | Limousin | A | 0.21 | 0.967 | GG | 0.62 |
|  |  | Simmental | A | 0.41 | 0.697 | GA | 0.48 |

| **SNP Name** | **SNP Reference** | **Breed** | **Minor Allele** | **Allele Frequency** | **Hardy Weinberg P value** | **Most Common Genotype** | **Genotype Frequency** |
| --- | --- | --- | --- | --- | --- | --- | --- |
| 423_24 | Werner *et al.,* (2004) | Aberdeen Angus | A | 0.16 | 0.548 | GG | 0.71 |
|  |  | Belgian Blue | A | 0.44 | 0.961 | GA | 0.49 |
|  |  | Charolais | A | 0.34 | 0.999 | GA | 0.45 |
|  |  | Holstein | G | 0.33 | 0.840 | AA | 0.45 |
|  |  | Limousin | A | 0.28 | 0.855 | GG | 0.52 |
|  |  | Simmental | G/A | 0.50 | 0.601 | GA | 0.50 |
| 425_2 | Werner *et al.,* (2004) | Aberdeen Angus | A | 0.05 | 0.564 | GG | 0.90 |
|  |  | Belgian Blue | A | 0.20 | 0.764 | GG | 0.64 |
|  |  | Charolais | A | 0.14 | 0.575 | GG | 0.74 |
|  |  | Holstein | A | 0.45 | 0.920 | AG | 0.50 |
|  |  | Limousin | A | 0.26 | 0.741 | GG | 0.55 |
|  |  | Simmental | A | 0.11 | 0.952 | GG | 0.79 |
| 022.t7 | Werner *et al.,* (2004) | Aberdeen Angus | A | 0.42 | 0.967 | AG | 0.49 |
|  |  | Belgian Blue | A | 0.49 | 0.891 | AG | 0.50 |
|  |  | Charolais | G | 0.29 | 0.999 | AA | 0.71 |
|  |  | Holstein | G | 0.48 | 0.945 | AG | 0.50 |
|  |  | Limousin | A | 0.42 | 0.460 | AG | 0.49 |
|  |  | Simmental | G | 0.47 | 0.481 | AG | 0.50 |
| 055.t7 | Werner *et al.,* (2004) | Aberdeen Angus | T | 0.20 | 0.968 | GG | 0.64 |
|  |  | Belgian Blue | T | 0.23 | 0.816 | GG | 0.59 |
|  |  | Charolais | T | 0.09 | 0.579 | GG | 0.83 |
|  |  | Holstein | T | 0.12 | 0.583 | GG | 0.77 |
|  |  | Limousin | T | 0.22 | 0.829 | GG | 0.61 |
|  |  | Simmental | T | 0.10 | 0.999 | GG | 0.81 |
| 058.sp6 | Werner *et al.,* (2004) | Aberdeen Angus | G | 0.41 | 0.967 | AG | 0.48 |
|  |  | Belgian Blue | G | 0.44 | 0.888 | AG | 0.49 |
|  |  | Charolais | G | 0.48 | 0.743 | AG | 0.50 |
|  |  | Holstein | G | 0.36 | 0.999 | AG | 0.46 |
|  |  | Limousin | G | 0.42 | 0.838 | AG | 0.49 |
|  |  | Simmental | G | 0.37 | 0.747 | AG | 0.47 |
| 436_C10 | Werner *et al.,* (2004) | Aberdeen Angus | C | 0.08 | 0.492 | TT | 0.84 |
|  |  | Belgian Blue | C | 0.27 | 0.888 | TT | 0.54 |
|  |  | Charolais | C | 0.05 | 0.999 | TT | 0.90 |
|  |  | Holstein | C | 0.24 | 0.761 | TT | 0.58 |
|  |  | Limousin | C | 0.26 | 0.969 | TT | 0.55 |
|  |  | Simmental | C | 0.20 | 0.351 | TT | 0.64 |
| Bulge105 | Werner *et al.,* (2004) | Aberdeen Angus | G | 0.07 | 0.999 | AA | 0.86 |
|  |  | Belgian Blue | G | 0.31 | 0.755 | AA | 0.47 |
|  |  | Charolais | G | 0.23 | 0.808 | AA | 0.60 |
|  |  | Holstein | G/A | 0.50 | 0.999 | GA | 0.50 |
|  |  | Limousin | G | 0.31 | 0.917 | AA | 0.47 |
|  |  | Simmental | G | 0.30 | 0.829 | AA | 0.49 |
| 105.sp6 | Werner *et al.,* (2004) | Aberdeen Angus | T | 0.08 | 0.999 | CC | 0.84 |
|  |  | Belgian Blue | T | 0.24 | 0.999 | CC | 0.58 |
|  |  | Charolais | T | 0.17 | 0.739 | CC | 0.69 |
|  |  | Holstein | T | 0.43 | 0.939 | CT | 0.49 |
|  |  | Limousin | T | 0.31 | 0.377 | CC | 0.48 |
|  |  | Simmental | T | 0.42 | 0.940 | CT | 0.49 |
| 039.t7 | Werner *et al.,* (2004) | Aberdeen Angus | C | 0.38 | 0.486 | CT | 0.47 |
|  |  | Belgian Blue | C | 0.21 | 0.968 | TT | 0.62 |
|  |  | Charolais | T | 0.42 | 0.812 | CT | 0.49 |
|  |  | Holstein | C | 0.32 | 0.628 | TT | 0.46 |
|  |  | Limousin | C | 0.29 | 0.728 | TT | 0.51 |
|  |  | Simmental | C | 0.30 | 0.999 | TT | 0.49 |

| **SNP Name** | **SNP Reference** | **Breed** | **Minor Allele** | **Allele Frequency** | **Hardy Weinberg P value** | **Most Common Genotype** | **Genotype Frequency** |
| --- | --- | --- | --- | --- | --- | --- | --- |
| 007.sp6 | Werner *et al.,* (2004) | Aberdeen Angus | A | 0.14 | 0.740 | GG | 0.74 |
|  |  | Belgian Blue | G | 0.49 | 0.891 | AG | 0.50 |
|  |  | Charolais | A | 0.30 | 0.849 | GG | 0.49 |
|  |  | Holstein | A | 0.36 | 0.999 | AG | 0.46 |
|  |  | Limousin | A | 0.24 | 0.888 | GG | 0.58 |
|  |  | Simmental | A | 0.36 | 0.857 | AG | 0.46 |
| Bulge 113 | Werner *et al.,* (2004) | Aberdeen Angus | C | 0.25 | 0.883 | TT | 0.56 |
|  |  | Belgian Blue | C | 0.17 | 0.548 | TT | 0.69 |
|  |  | Charolais | C | 0.24 | 0.999 | TT | 0.58 |
|  |  | Holstein | T | 0.34 | 0.333 | CT | 0.45 |
|  |  | Limousin | C | 0.16 | 0.999 | TT | 0.70 |
|  |  | Simmental | C | 0.24 | 0.767 | TT | 0.58 |
| 048.sp6 | Werner *et al.,* (2004) | Aberdeen Angus | T | 0.45 | 0.478 | GT | 0.50 |
|  |  | Belgian Blue | T | 0.20 | 0.999 | GG | 0.64 |
|  |  | Charolais | T | 0.44 | 0.999 | GT | 0.49 |
|  |  | Holstein | T | 0.26 | 0.253 | GG | 0.55 |
|  |  | Limousin | T | 0.39 | 0.942 | GT | 0.48 |
|  |  | Simmental | G | 0.36 | 0.999 | GT | 0.46 |
| 070.t7 | Werner *et al.,* (2004) | Aberdeen Angus | C | 0.18 | 0.999 | AA | 0.67 |
|  |  | Belgian Blue | C | 0.18 | 0.593 | AA | 0.67 |
|  |  | Charolais | C | 0.38 | 0.999 | AC | 0.47 |
|  |  | Holstein | C | 0.43 | 0.957 | AC | 0.49 |
|  |  | Limousin | A | 0.43 | 0.999 | AC | 0.49 |
|  |  | Simmental | C | 0.31 | 0.966 | AA | 0.47 |
| 090.t7 | Werner *et al.,* (2004) | Aberdeen Angus | A | 0.24 | 0.999 | GG | 0.58 |
|  |  | Belgian Blue | G | 0.46 | 0.999 | AG | 0.50 |
|  |  | Charolais | A | 0.45 | 0.756 | AG | 0.50 |
|  |  | Holstein | A | 0.46 | 0.999 | AG | 0.50 |
|  |  | Limousin | A/G | 0.50 | 0.896 | AG | 0.50 |
|  |  | Simmental | G | 0.28 | 0.888 | AA | 0.52 |
| MBS029-1 | Heaton *et al.,* (2002) | Aberdeen Angus | G | 0.24 | 0.782 | AA | 0.58 |
|  |  | Belgian Blue | G | 0.31 | 0.615 | AA | 0.47 |
|  |  | Charolais | G | 0.32 | 0.250 | AA | 0.46 |
|  |  | Holstein | A | 0.40 | 0.404 | AG | 0.48 |
|  |  | Limousin | G | 0.47 | 0.641 | AG | 0.50 |
|  |  | Simmental | A | 0.40 | 0.753 | AG | 0.48 |
| MBS042-1 | Heaton *et al.,* (2002) | Aberdeen Angus | G | 0.45 | 0.317 | AG | 0.50 |
|  |  | Belgian Blue | A | 0.30 | 0.762 | GG | 0.49 |
|  |  | Charolais | A | 0.25 | 0.999 | GG | 0.56 |
|  |  | Holstein | G | 0.44 | 0.999 | AG | 0.50 |
|  |  | Limousin | A | 0.40 | 0.656 | AG | 0.48 |
|  |  | Simmental | A | 0.05 | 0.513 | GG | 0.90 |
| MBS048-1 | Heaton *et al.,* (2002) | Aberdeen Angus | G | 0.41 | 0.967 | CG | 0.48 |
|  |  | Belgian Blue | G | 0.23 | 0.999 | CC | 0.60 |
|  |  | Charolais | C | 0.27 | 0.683 | GG | 0.53 |
|  |  | Holstein | C | 0.36 | 0.999 | CG | 0.46 |
|  |  | Limousin | C | 0.20 | 0.555 | GG | 0.64 |
|  |  | Simmental | C | 0.27 | 0.886 | GG | 0.53 |
| MBS007-1 | Heaton *et al.,* (2002) | Aberdeen Angus | A | 0.24 | 0.782 | CC | 0.58 |
|  |  | Belgian Blue | A | 0.23 | 0.459 | CC | 0.60 |
|  |  | Charolais | A | 0.06 | 0.999 | CC | 0.88 |
|  |  | Holstein | A | 0.10 | 0.579 | CC | 0.81 |
|  |  | Limousin | A | 0.08 | 0.999 | CC | 0.85 |
|  |  | Simmental | A | 0.41 | 0.749 | AC | 0.48 |

| **SNP Name** | **SNP Reference** | **Breed** | **Minor Allele** | **Allele Frequency** | **Hardy Weinberg P value** | **Most Common Genotype** | **Genotype Frequency** |
| --- | --- | --- | --- | --- | --- | --- | --- |
| MBS030-1 | Heaton *et al.,* (2002) | Aberdeen Angus | A | 0.01 | 0.999 | GG | 0.98 |
|  |  | Belgian Blue | A | 0.10 | 0.583 | GG | 0.81 |
|  |  | Charolais | A | 0.25 | 0.767 | GG | 0.56 |
|  |  | Holstein | A | 0.31 | 0.835 | GG | 0.48 |
|  |  | Limousin | A | 0.26 | 0.888 | GG | 0.55 |
|  |  | Simmental | A | 0.18 | 0.592 | GG | 0.67 |
| MBS043-1 | Heaton *et al.,* (2002) | Aberdeen Angus | G | 0.39 | 0.580 | GT | 0.48 |
|  |  | Belgian Blue | T | 0.35 | 0.575 | GT | 0.46 |
|  |  | Charolais | T | 0.41 | 0.697 | GT | 0.48 |
|  |  | Holstein | G | 0.47 | 0.868 | GT | 0.50 |
|  |  | Limousin | T | 0.42 | 0.738 | GT | 0.49 |
|  |  | Simmental | T | 0.34 | 0.914 | GT | 0.44 |
| AH2-5 | Heaton *et al.,* (2002) | Aberdeen Angus | C | 0.17 | 0.758 | TT | 0.69 |
|  |  | Belgian Blue | C | 0.34 | 0.999 | CT | 0.45 |
|  |  | Charolais | C | 0.38 | 0.566 | CT | 0.47 |
|  |  | Holstein | T | 0.45 | 0.867 | CT | 0.50 |
|  |  | Limousin | T | 0.16 | 0.548 | CC | 0.71 |
|  |  | Simmental | C | 0.20 | 0.868 | TT | 0.64 |
| MBS044-1 | Heaton *et al.,* (2002) | Aberdeen Angus | A | 0.42 | 0.787 | AG | 0.49 |
|  |  | Belgian Blue | A | 0.26 | 0.512 | GG | 0.55 |
|  |  | Charolais | A | 0.21 | 0.473 | GG | 0.62 |
|  |  | Holstein | A | 0.09 | 0.467 | GG | 0.83 |
|  |  | Limousin | A | 0.35 | 0.931 | AG | 0.46 |
|  |  | Simmental | A | 0.25 | 0.772 | GG | 0.56 |
| MBS031-1 | Heaton *et al.,* (2002) | Aberdeen Angus | T | 0.37 | 0.999 | CT | 0.47 |
|  |  | Belgian Blue | T | 0.07 | 0.999 | CC | 0.86 |
|  |  | Charolais | T | 0.09 | 0.741 | CC | 0.83 |
|  |  | Holstein | T | 0.21 | 0.592 | CC | 0.63 |
|  |  | Limousin | T | 0.43 | 0.999 | CT | 0.49 |
|  |  | Simmental | T | 0.09 | 0.573 | CC | 0.83 |
| AH8-4 | Heaton *et al.,* (2002) | Aberdeen Angus | A | 0.42 | 0.999 | AG | 0.49 |
|  |  | Belgian Blue | A | 0.39 | 0.877 | AG | 0.48 |
|  |  | Charolais | G | 0.21 | 0.826 | AA | 0.63 |
|  |  | Holstein | A | 0.38 | 0.857 | AG | 0.47 |
|  |  | Limousin | A | 0.39 | 0.968 | AG | 0.48 |
|  |  | Simmental | A | 0.28 | 0.966 | GG | 0.52 |
| MBS015-1 | Heaton *et al.,* (2002) | Aberdeen Angus | T | 0.46 | 0.370 | CT | 0.50 |
|  |  | Belgian Blue | C/T | 0.50 | 0.819 | CT | 0.50 |
|  |  | Charolais | C | 0.30 | 0.999 | TT | 0.49 |
|  |  | Holstein | T | 0.41 | 0.542 | CT | 0.48 |
|  |  | Limousin | C | 0.49 | 0.500 | CT | 0.50 |
|  |  | Simmental | C | 0.36 | 0.964 | CT | 0.46 |
| AH25-1 | Heaton *et al.,* (2002) | Aberdeen Angus | A | 0.29 | 0.841 | GG | 0.50 |
|  |  | Belgian Blue | A | 0.46 | 0.392 | AG | 0.50 |
|  |  | Charolais | G | 0.26 | 0.472 | AA | 0.55 |
|  |  | Holstein | G/A | 0.50 | 0.871 | AG | 0.50 |
|  |  | Limousin | G | 0.23 | 0.999 | AA | 0.60 |
|  |  | Simmental | G | 0.33 | 0.915 | AA | 0.45 |
| MBS046-1 | Heaton *et al.,* (2002) | Aberdeen Angus | C | 0.32 | 0.773 | TT | 0.46 |
|  |  | Belgian Blue | C | 0.12 | 0.586 | TT | 0.78 |
|  |  | Charolais | C | 0.27 | 0.683 | TT | 0.54 |
|  |  | Holstein | C | 0.34 | 0.628 | CT | 0.45 |
|  |  | Limousin | C | 0.20 | 0.828 | TT | 0.64 |
|  |  | Simmental | C | 0.10 | 0.560 | TT | 0.81 |

| **SNP Name** | **SNP Reference** | **Breed** | **Minor Allele** | **Allele Frequency** | **Hardy Weinberg P value** | **Most Common Genotype** | **Genotype Frequency** |
| --- | --- | --- | --- | --- | --- | --- | --- |
| MBS018-1 | Heaton *et al.,* (2002) | Aberdeen Angus | T | 0.16 | 0.999 | GG | 0.71 |
|  |  | Belgian Blue | T | 0.43 | 0.769 | GT | 0.49 |
|  |  | Charolais | T | 0.23 | 0.966 | GG | 0.60 |
|  |  | Holstein | G | 0.20 | 0.835 | TT | 0.64 |
|  |  | Limousin | T | 0.18 | 0.758 | GG | 0.67 |
|  |  | Simmental | T | 0.47 | 0.999 | GT | 0.50 |
| MBS025-1 | Heaton *et al.,* (2002) | Aberdeen Angus | C | 0.44 | 0.691 | CT | 0.50 |
|  |  | Belgian Blue | C | 0.39 | 0.877 | CT | 0.48 |
|  |  | Charolais | T | 0.15 | 0.590 | CC | 0.72 |
|  |  | Holstein | T | 0.33 | 0.840 | CC | 0.45 |
|  |  | Limousin | T | 0.22 | 0.968 | CC | 0.61 |
|  |  | Simmental | T | 0.10 | 0.600 | CC | 0.81 |
| MBS035-1 | Heaton *et al.,* (2002) | Aberdeen Angus | A | 0.33 | 0.593 | GG | 0.45 |
|  |  | Belgian Blue | G | 0.22 | 0.727 | AA | 0.61 |
|  |  | Charolais | A | 0.39 | 0.264 | AG | 0.48 |
|  |  | Holstein | G | 0.27 | 0.431 | AA | 0.53 |
|  |  | Limousin | G | 0.39 | 0.942 | AG | 0.48 |
|  |  | Simmental | A | 0.23 | 0.768 | GG | 0.60 |
| MBS028-1 | Heaton *et al.,* (2002) | Aberdeen Angus | A | 0.31 | 0.915 | GG | 0.48 |
|  |  | Belgian Blue | G | 0.37 | 0.875 | GA | 0.47 |
|  |  | Charolais | G | 0.33 | 0.608 | AA | 0.45 |
|  |  | Holstein | A | 0.34 | 0.824 | GA | 0.45 |
|  |  | Limousin | A | 0.33 | 0.433 | GG | 0.45 |
|  |  | Simmental | G | 0.29 | 0.886 | AA | 0.51 |
| MBS040-1 | Heaton *et al.,* (2002) | Aberdeen Angus | T | 0.35 | 0.970 | CT | 0.46 |
|  |  | Belgian Blue | C | 0.43 | 0.884 | CT | 0.49 |
|  |  | Charolais | T | 0.48 | 0.952 | CT | 0.50 |
|  |  | Holstein | T | 0.28 | 0.999 | CC | 0.52 |
|  |  | Limousin | C | 0.47 | 0.893 | CT | 0.50 |
|  |  | Simmental | C | 0.35 | 0.853 | CT | 0.46 |
| MBS041-1 | Heaton *et al.,* (2002) | Aberdeen Angus | T | 0.24 | 0.782 | CC | 0.58 |
|  |  | Belgian Blue | T | 0.22 | 0.307 | CC | 0.61 |
|  |  | Charolais | T | 0.39 | 0.377 | CT | 0.48 |
|  |  | Holstein | T | 0.04 | 0.999 | CC | 0.91 |
|  |  | Limousin | T | 0.27 | 0.214 | CC | 0.54 |
|  |  | Simmental | T | 0.30 | 0.102 | CC | 0.49 |
| MBS021 -1 | Heaton *et al.,* (2002) | Aberdeen Angus | T | 0.49 | 0.171 | TG | 0.50 |
|  |  | Belgian Blue | T | 0.44 | 0.620 | TG | 0.49 |
|  |  | Charolais | G | 0.42 | 0.309 | TG | 0.49 |
|  |  | Holstein | G | 0.46 | 0.276 | TG | 0.50 |
|  |  | Limousin | G | 0.46 | 0.963 | TG | 0.50 |
|  |  | Simmental | G | 0.33 | 0.608 | TT | 0.45 |
| MBS033-1 | Heaton *et al.,* (2002) | Aberdeen Angus | G | 0.20 | 0.826 | AA | 0.64 |
|  |  | Belgian Blue | G | 0.31 | 0.755 | AA | 0.47 |
|  |  | Charolais | A | 0.23 | 0.200 | GG | 0.60 |
|  |  | Holstein | G | 0.29 | 0.963 | AA | 0.50 |
|  |  | Limousin | A | 0.32 | 0.872 | GG | 0.46 |
|  |  | Simmental | A | 0.17 | 0.999 | GG | 0.69 |
| MBS054-1 | Heaton *et al.,* (2002) | Aberdeen Angus | A | 0.41 | 0.949 | AC | 0.48 |
|  |  | Belgian Blue | A | 0.20 | 0.312 | CC | 0.64 |
|  |  | Charolais | A/C | 0.50 | 0.885 | AC | 0.50 |
|  |  | Holstein | A | 0.36 | 0.999 | AC | 0.46 |
|  |  | Limousin | C | 0.32 | 0.539 | AA | 0.46 |
|  |  | Simmental | A | 0.45 | 0.751 | AC | 0.50 |

| **SNP Name** | **SNP Reference** | **Breed** | **Minor Allele** | **Allele Frequency** | **Hardy Weinberg P value** | **Most Common Genotype** | **Genotype Frequency** |
| --- | --- | --- | --- | --- | --- | --- | --- |
| 16_2 | Werner *et al.,* (2004) | Aberdeen Angus | Monomorphic G | 1.00 | N/A | GG | 1.00 |
|  |  | Belgian Blue | Monomorphic G | 1.00 | N/A | GG | 1.00 |
|  |  | Charolais | A | 0.08 | 0.999 | GG | 0.85 |
|  |  | Holstein | A | 0.26 | 0.661 | GG | 0.55 |
|  |  | Limousin | A | 0.05 | 0.999 | GG | 0.90 |
|  |  | Simmental | A | 0.25 | 0.999 | GG | 0.56 |
| 448_67 | Werner *et al.,* (2004) | Aberdeen Angus | C | 0.03 | 0.999 | TT | 0.93 |
|  |  | Belgian Blue | C | 0.04 | 0.601 | TT | 0.92 |
|  |  | Charolais | C | 0.11 | 0.946 | TT | 0.79 |
|  |  | Holstein | C | 0.36 | 0.527 | CT | 0.46 |
|  |  | Limousin | C | 0.14 | 0.590 | TT | 0.74 |
|  |  | Simmental | C | 0.02 | 0.999 | TT | 0.96 |
| 487_67 | Werner *et al.,* (2004) | Aberdeen Angus | G | 0.36 | 0.884 | GA | 0.46 |
|  |  | Belgian Blue | A | 0.10 | 0.947 | GG | 0.81 |
|  |  | Charolais | A | 0.12 | 0.586 | GG | 0.77 |
|  |  | Holstein | A | 0.40 | 0.929 | GA | 0.48 |
|  |  | Limousin | A | 0.15 | 0.999 | GG | 0.72 |
|  |  | Simmental | A | 0.09 | 0.573 | GG | 0.83 |
| Bulge 101 | Werner *et al.,* (2004) | Aberdeen Angus | T | 0.13 | 0.270 | CC | 0.76 |
|  |  | Belgian Blue | T | 0.09 | 0.999 | CC | 0.83 |
|  |  | Charolais | T | 0.03 | 0.999 | CC | 0.94 |
|  |  | Holstein | T | 0.48 | 0.722 | CT | 0.50 |
|  |  | Limousin | T | 0.05 | 0.999 | CC | 0.90 |
|  |  | Simmental | T | 0.08 | 0.922 | CC | 0.85 |
| MBS047-1 | Heaton *et al.,* (2002) | Aberdeen Angus | T | 0.30 | 0.916 | GG | 0.49 |
|  |  | Belgian Blue | T | 0.17 | 0.548 | GG | 0.69 |
|  |  | Charolais | T | 0.11 | 0.578 | GG | 0.80 |
|  |  | Holstein | T | 0.12 | 0.999 | GG | 0.77 |
|  |  | Limousin | T | 0.05 | 0.999 | GG | 0.90 |
|  |  | Simmental | T | 0.30 | 0.829 | GG | 0.49 |
| 454G_11 | Werner *et al.,* (2004) | Aberdeen Angus | G | 0.13 | 0.590 | CC | 0.75 |
|  |  | Belgian Blue | G | 0.20 | 0.965 | CC | 0.64 |
|  |  | Charolais | G | 0.10 | 0.999 | CC | 0.81 |
|  |  | Holstein | G | 0.26 | 0.806 | CC | 0.55 |
|  |  | Limousin | G | 0.16 | 0.548 | CC | 0.70 |
|  |  | Simmental | G | 0.06 | 0.999 | CC | 0.88 |

Additional file 1 Table S1 – SNP allele frequencies, Hardy Weinberg Equilibrium chi squared p values and most common genotype frequencies.
