# Supplementary material for: Compilation of a panel of informative single nucleotide polymorphisms for bovine identification in the Northern Irish cattle population
Source: BMC Genet. 2010 Jan 25;11:5. doi: 10.1186/1471-2156-11-5 (PMC2826282; doi:10.1186/1471-2156-11-5)
Supplement: Additional file 2 — STR allele frequency data. All STR allele frequency data for the NI cattle population representative random sampling are contained in this table along with Hardy Weinberg chi square p values, most common genotypes and most common genotype frequency. [file 1471-2156-11-5-S2.DOC]

| **STR Locus Identifier** | **STR alleles (bp)** | **Allele Frequencies** | **Hardy Weinberg Statistic** | **Most Common Genotype** | **Genotype Frequency** |
| --- | --- | --- | --- | --- | --- |
| BM1824 | 178 | 0.2309 | 0.975 | 182 / 188 | 0.15 |
|  | 180 | 0.1899 |  |  |  |
|  | 182 | 0.3169 |  |  |  |
|  | 184 | 0.0014 |  |  |  |
|  | 188 | 0.2418 |  |  |  |
|  | 190 | 0.0191 |  |  |  |
| BM2113 | 121 | 0.0068 | 0.611 | 135 / 137 | 0.09 |
|  | 125 | 0.0847 |  |  |  |
|  | 127 | 0.0820 |  |  |  |
|  | 131 | 0.1189 |  |  |  |
|  | 133 | 0.1489 |  |  |  |
|  | 135 | 0.2131 |  |  |  |
|  | 137 | 0.2090 |  |  |  |
|  | 139 | 0.1148 |  |  |  |
|  | 141 | 0.0164 |  |  |  |
|  | 143 | 0.0055 |  |  |  |
| ETH10 | 209 | 0.0041 | 0.817 | 217 / 219 | 0.23 |
|  | 213 | 0.0191 |  |  |  |
|  | 215 | 0.0369 |  |  |  |
|  | 217 | 0.4208 |  |  |  |
|  | 219 | 0.2678 |  |  |  |
|  | 221 | 0.1284 |  |  |  |
|  | 223 | 0.0697 |  |  |  |
|  | 225 | 0.0533 |  |  |  |
| ETH225 | 140 | 0.2077 | 0.683 | 148 / 150 | 0.18 |
|  | 142 | 0.0082 |  |  |  |
|  | 144 | 0.0779 |  |  |  |
|  | 146 | 0.0669 |  |  |  |
|  | 148 | 0.2664 |  |  |  |
|  | 150 | 0.3443 |  |  |  |
|  | 152 | 0.0287 |  |  |  |

| **STR Locus Identifier** | **STR alleles (bp)** | **Allele Frequencies** | **Hardy Weinberg Statistic** | **Most Common Genotype** | **Genotype Frequency** |
| --- | --- | --- | --- | --- | --- |
| ETH3 | 107 | 0.0232 | 0.557 | 117 / 117 | 0.28 |
|  | 117 | 0.5273 |  |  |  |
|  | 119 | 0.1052 |  |  |  |
|  | 121 | 0.0642 |  |  |  |
|  | 123 | 0.0164 |  |  |  |
|  | 125 | 0.1393 |  |  |  |
|  | 127 | 0.0833 |  |  |  |
|  | 129 | 0.0383 |  |  |  |
|  | 131 | 0.0027 |  |  |  |
| INRA23 | 198 | 0.0205 | 0.754 | 206 / 214 | 0.14 |
|  | 200 | 0.0779 |  |  |  |
|  | 202 | 0.0779 |  |  |  |
|  | 204 | 0.0055 |  |  |  |
|  | 206 | 0.2814 |  |  |  |
|  | 208 | 0.1516 |  |  |  |
|  | 210 | 0.0724 |  |  |  |
|  | 212 | 0.0423 |  |  |  |
|  | 214 | 0.2404 |  |  |  |
|  | 216 | 0.0164 |  |  |  |
|  | 218 | 0.0137 |  |  |  |
| SPS115 | 240 | 0.0027 | 0.962 | 248 / 248 | 0.34 |
|  | 248 | 0.5861 |  |  |  |
|  | 250 | 0.0014 |  |  |  |
|  | 252 | 0.0833 |  |  |  |
|  | 254 | 0.1079 |  |  |  |
|  | 256 | 0.0902 |  |  |  |
|  | 258 | 0.0205 |  |  |  |
|  | 260 | 0.1066 |  |  |  |
|  | 264 | 0.0014 |  |  |  |

| **STR Locus Identifier** | **STR alleles (bp)** | **Allele Frequencies** | **Hardy Weinberg Statistic** | **Most Common Genotype** | **Genotype Frequency** |
| --- | --- | --- | --- | --- | --- |
| TGLA122 | 139 | 0.0027 | 0.736 | 143 / 151 | 0.18 |
|  | 141 | 0.0301 |  |  |  |
|  | 143 | 0.3579 |  |  |  |
|  | 147 | 0.0246 |  |  |  |
|  | 149 | 0.0587 |  |  |  |
|  | 151 | 0.2445 |  |  |  |
|  | 153 | 0.0765 |  |  |  |
|  | 155 | 0.0055 |  |  |  |
|  | 157 | 0.0150 |  |  |  |
|  | 159 | 0.0014 |  |  |  |
|  | 161 | 0.0519 |  |  |  |
|  | 163 | 0.0383 |  |  |  |
|  | 167 | 0.0014 |  |  |  |
|  | 169 | 0.0068 |  |  |  |
|  | 171 | 0.0246 |  |  |  |
|  | 173 | 0.0082 |  |  |  |
|  | 175 | 0.0014 |  |  |  |
|  | 177 | 0.0055 |  |  |  |
|  | 179 | 0.0191 |  |  |  |
|  | 181 | 0.0027 |  |  |  |
|  | 183 | 0.0232 |  |  |  |
| TGLA126 | 113 | 0.0191 | 0.773 | 115 / 115 | 0.26 |
|  | 115 | 0.5082 |  |  |  |
|  | 117 | 0.2063 |  |  |  |
|  | 119 | 0.0219 |  |  |  |
|  | 121 | 0.0574 |  |  |  |
|  | 123 | 0.1844 |  |  |  |
|  | 125 | 0.0027 |  |  |  |

| **STR Locus Identifier** | **STR alleles (bp)** | **Allele Frequencies** | **Hardy Weinberg Statistic** | **Most Common Genotype** | **Genotype Frequency** |
| --- | --- | --- | --- | --- | --- |
| TGLA 227 | 77 | 0.0219 | 0.832 | 81 / 89 | 0.09 |
|  | 79 | 0.0082 |  |  |  |
|  | 81 | 0.2131 |  |  |  |
|  | 83 | 0.0847 |  |  |  |
|  | 85 | 0.0246 |  |  |  |
|  | 87 | 0.0478 |  |  |  |
|  | 89 | 0.2172 |  |  |  |
|  | 91 | 0.1325 |  |  |  |
|  | 93 | 0.0697 |  |  |  |
|  | 95 | 0.0041 |  |  |  |
|  | 97 | 0.1557 |  |  |  |
|  | 99 | 0.0068 |  |  |  |
|  | 101 | 0.0137 |  |  |  |
| TGLA53 | 154 | 0.0792 | 0.999 | 160 / 168 | 0.06 |
|  | 158 | 0.0505 |  |  |  |
|  | 160 | 0.1976 |  |  |  |
|  | 162 | 0.1134 |  |  |  |
|  | 164 | 0.0574 |  |  |  |
|  | 166 | 0.0669 |  |  |  |
|  | 168 | 0.1407 |  |  |  |
|  | 170 | 0.0888 |  |  |  |
|  | 172 | 0.0519 |  |  |  |
|  | 174 | 0.0082 |  |  |  |
|  | 176 | 0.0724 |  |  |  |
|  | 178 | 0.0068 |  |  |  |
|  | 180 | 0.0027 |  |  |  |
|  | 182 | 0.0191 |  |  |  |
|  | 184 | 0.0164 |  |  |  |
|  | 186 | 0.0219 |  |  |  |
|  | 188 | 0.0014 |  |  |  |
|  | 190 | 0.0055 |  |  |  |

Additional file 2 Table S2 - STR allele frequencies, Hardy Weinberg Equilibrium chi squared p values and most common genotype frequencies.
